# Validation of AmpliSeq NGS Panel for *BRCA1* and *BRCA2* Variant Detection in Canine Formalin-Fixed Paraffin-Embedded Mammary Tumors

**DOI:** 10.3390/life12060851

**Published:** 2022-06-07

**Authors:** Daniela Di Giacomo, Marco Di Domenico, Sabrina Vanessa Patrizia Defourny, Daniela Malatesta, Giovanni Di Teodoro, Michele Martino, Antonello Viola, Nicola D’Alterio, Cesare Cammà, Paola Modesto, Antonio Petrini

**Affiliations:** 1 Istituto Zooprofilattico Sperimentale dell’Abruzzo e del Molise “G. Caporale”, Campo Boario, 64100 Teramo, Italy; m.didomenico@izs.it (M.D.D.); s.defourny@izs.it (S.V.P.D.); d.malatesta@izs.it (D.M.); g.diteodoro@izs.it (G.D.T.); m.martino@izs.it (M.M.); n.dalterio@izs.it (N.D.); c.camma@izs.it (C.C.); a.petrini@izs.it (A.P.); 2 National Reference Center for Whole Genome Sequencing of Microbial Pathogens: Database and Bioinformatic Analysis, Istituto Zooprofilattico Sperimentale dell’Abruzzo e del Molise, Campo Boario, 64100 Teramo, Italy; 3 Veterinary Practitioner, Centro Veterinario Nova Julia, Via Galileo Galilei 177, 64021 Giulianova, Italy; antonello.viola@aslteramo.it; 4 Istituto Zooprofilattico Sperimentale del Piemonte, Liguria e Valle d’Aosta, Via Bologna 148, 10154 Torino, Italy; paola.modesto@izsto.it

**Keywords:** CMTs, FFPE, validation, repeatability, dog tumors

## Abstract

Mammary carcinomas are the most common neoplasms observed in women and in female dogs. Canine mammary tumors show epidemiological, clinical, genetic, and prognostic characteristics comparable to human breast cancers. The recent introduction of next generation sequencing (NGS) technologies has greatly improved research and diagnostics for humans, while these new tools still need to be implemented in animal models. In this study we developed and validated an AmpliSeq Panel assay for the identification of *BRCA* variants in twenty-two different dogs. The amplicon mean coverage was 5499× and uniformity was higher than 98% in all samples. The results of germline single nucleotide variants (SNVs) and insertions/deletions (INDELs) were fully concordant regardless of the types of samples considered (blood, fresh and FFPE tissues). Moreover, despite the high DNA degradation observed in older FFPE blocks (>5 years), the assay allowed full coverage of all amplicons for downstream analyses. We consider the NGS panel developed in this study as a useful tool for expanding information on *BRCA* genes in the veterinary field and for human health from a comparative oncology perspective.

## 1. Introduction

*BRCA1* and *BRCA2* are tumor suppressor genes that have been associated with increased risk, mainly for breast and ovarian human cancers, when carrying specific mutations. These genes are also involved in canine mammary tumors (CMTs), which are the most common neoplasms in female animals, representing a major health threat also for dogs [1].

Similarities in the biological, epidemiological, and genetic characteristics of canine and human mammary tumors suggested that dogs may provide a useful model for human breast cancer research. Humans and dogs share over 80% of the aminoacidic residues of *BRCA* genes [2]. Moreover, the clinically common traits of human and dog breast cancer include spontaneous tumor incidence, onset age, hormonal etiology, and the identical course of the disease. In addition, canine tumors also exhibit the principal pathologic features of human cancers, including a long-term oncogenic setting, intratumoral heterogeneity, acquired resistance to treatments, tumor size, clinical stage, lymph node invasion and distant metastases [3,4]. 

The recent introduction of next-generation sequencing (NGS) is changing the genetic analysis and research of human *BRCA* due to its high throughput and cost-effectiveness [5,6,7]. These new tools allow researchers to detect already known SNVs, but also make possible the discovery of new variants within these two large genes. 

The most common technique for downstream diagnosis, classification, and storage of cancer tissues in many veterinary pathology laboratories is formalin-fixed, paraffin-embedded (FFPE). The use of this technique has also intensified to enlarge the biobanks. All this has led to a significant increase in FFPE samples that could be available for further analysis, such as retrospective investigations. FFPE blocks also provide useful information on histotypes, in addition to the presence of precancerous lesions. Such data, combined with other techniques such as immunohistochemistry and NGS, can complete the scenario of specific lesions. 

In human medicine, many studies have shown the effectiveness of using the NGS technique applied to FFPE tissues [8,9]; however, despite this, in veterinary medicine there are few reports that use this technique, many of which are focused on etiological agent detection [10]. An AmpliSeq panel for *BRCA1* and *BRCA2* has recently been published for cats [11], while the great potential of NGS is not yet explored for *BRCA* research and diagnostics in dogs. To date, studies on dog *BRCA* mutations and the possible association with mammary cancer are very limited. Unfortunately, most investigations have focused on highly conserved regions only, which often represent sites of interaction with the RAD51 protein [12,13,14].

Aiming to fill in this gap, we present here the development and validation of a new AmpliSeq *BRCA* Panel assay on an Illumina platform for the identification of variants across *BRCA1* and *BRCA2* genes in dogs. Rather than the differentiation between somatic or germline variations introduced in neoplastic tissues and those inherited, this study focuses instead on the analysis of FFPE blocks. It is also worth considering that this method may open a new window for the retrospective analysis of historical samples. 

Finally, we compared variants found in dogs with those already described in humans to evaluate the possible clinical effects and a new strategy for classification in canine samples. 

## 2. Materials and Methods

### 2.1. Sample Selection

Twenty-two FFPE tissue samples from pathological (n. 20) and normal (n. 2) canine mammary tissues were retrieved from the archives of the Diagnostic Department of Istituto Zooprofilattico Sperimentale dell’Abruzzo e del Molise (Table 1). Seven out of twenty mammary tumors were submitted to the laboratory immediately after surgery, together with a 1 mL blood sample collected in EDTA. These neoplastic specimens were sectioned in half: one part was fixed in 10% buffered formalin for 48 h, processed and embedded in paraffin wax, and the other part was frozen at −80 °C. From these seven animals (ID: 1, 2, 3, 4, 7, 8, 9), we sampled blood, FFPE blocks and fresh tissues. Normal mammary gland tissues were collected during routine necropsies from female dogs who died from causes unrelated to oncologic diseases. Written consent for specimen collection and analysis was provided by each owner. Medical history and anamnesis, including breed, age and sex, were taken when available. FFPE samples were also grouped as old (2014–2016), medium (2017–2019) and recent (2020–2021), depending on the time of storage. 

### 2.2. DNA Extraction

Genomic DNA was extracted from blood, fresh tissues, and FFPE specimens. More precisely, DNA from blood samples (200 µL) and fresh tissues (25 mg) were extracted by means of the Maxwell 16 Blood DNA Purification Kit (Promega, Madison, CA, USA) and the Maxwell 16 Tissue DNA Purification Kit (Promega), respectively. FFPE samples were extracted using the Maxwell^®^ RSC DNA FFPE Kit (Promega) with a slight modification to the protocol at point 7 where the samples were incubated on a heat block at 65 °C overnight instead of 80 °C for 4 h. Cartridges provided by the three different kits were loaded on the Maxwell 16 Instrument (Promega). Quality control was performed on DNA by the Agilent 4200 TapeStation System (Agilent Technologies, Santa Clara, CA, USA) using the Genomic DNA ScreenTape and Genomic DNA reagents (Agilent Technologies). DNA was also quantified by the Qubit 2.0 Fluorometer (Thermo Fisher Scientific, Waltham, MA, USA) and then diluted to 4 ng/µL using Low TE buffer (10 mM Tris-HCl (pH 8.0) + 0.1 mM EDTA) for library preparation.

### 2.3. Sequencing and Data Analysis

A total of 197 custom amplicons (Supplementary Materials) were used for specific amplification of 19,804 bp *BRCA1* and *BRCA2* exonic regions of *Canis familiaris* (UCSC canFam3). The amplicon average length was 162 bp, with a maximum length of 175 bp. Libraries were prepared by means of the AmpliSeq Library PLUS (Illumina, San Diego, CA, USA) and AmpliSeq UD Indexes for Illumina (IDT, Coralville, IA, USA), following the protocol described in the AmpliSeq for Illumina On-demand, Custom, and Community Panels Reference Guide (Document # 1000000036408 v09). Quality control of the individual libraries (size and concentration) was fulfilled by the Agilent 4200 TapeStation System (Agilent Technologies), using the High Sensitivity D5000 ScreenTape and reagents (Agilent Technologies), and Qubit 2.0 Fluorometer (Thermo Fisher Scientific). Manually normalized libraries were then pooled, diluted to 1.2 pM and loaded in the MiniSeq Mid Output reagent cartridge (Illumina). Sequencing was performed by the Illumina MiniSeq Sequencer setting 300 cycles (150 bp paired-end reads), followed by primary analysis performed by the on-instrument software SCS/RTA (Illumina). Secondary analysis was accomplished by the DNA Amplicon workflow available on Local Run Manager Version 2.4.1 (Illumina, San Diego, CA, USA), using the manifest file (IAA19398_179_manifest.txt) (Supplementary Materials). Analyses were performed by using the software aligner BWA-MEM Whole-Genome (v0.7.9a-isis-1.0.2), Pisces Variant Caller (v5.2.9.23), Bam Metrics (v0.0.22) and SAMtools (v0.1.19-isis-1.0.3), all provided by Illumina (San Diego, CA, USA). Only variants with quality values 100 and filter “pass” were considered for the analysis. Finally, tertiary analysis was established by using both the Genome Browser, available online at https://genome.ucsc.edu/ (accessed on 27 May 2022), on CanFam3/CanFam3.1 genome, and Alamut Plus software v.1.0 (SOPHiA GENETICS SA, Lausanne, VD, CH), in order to gain biological significance for *BRCA1*/*BRCA2* variants from dog versus human comparisons. Statistical analysis was completed by the Kruskal–Wallis non-parametric test. 

## 3. Results

### 3.1. DNA Quality

**Figure 1:**
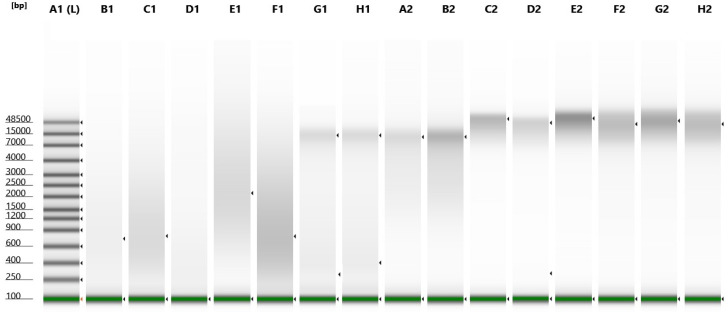
Three samples per group were randomly used. A1: Ladder, B1-C1-D1: FFPE old samples (2014 – 2016), E1-F1-G1: FFPE medium samples (2017–2019), H1-A2-B2: FFPE recent samples (2020 – 2021), C2-D2-E2: Blood samples, F2-G2-H2: Fresh tissue samples.

Results from Qubit quantification showed a DNA concentration higher than the input required by the protocol (4 ng/µL) in all the samples considered in this study. DNA integrity (DIN) evidenced different grades of fragmentation according to the time of storage of FFPE samples and to the different matrices considered (FFPE, blood, fresh tissue). As expected, the highest level of degradation was observed for FFPE samples and, within them, for those stored for a larger period (Table 2, Figure 1).

### 3.2. Amplicon Coverage and Variants Found

**Figure 2:**
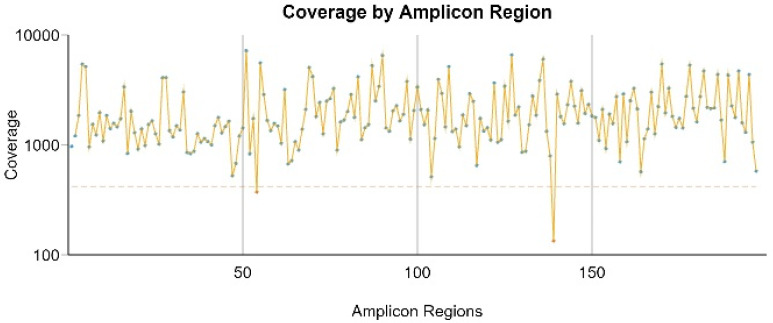
Details of the amplicon coverage. The 197 regions are uniformly covered (green dot), except for 2 amplicons (red dot). The threshold (dotted red line) marks coverage values above 20% of the mean value (Pct > 0.2 mean).

The amplicon mean (deep) coverage was 5499×, and a mean of 1.2 M passing filter reads (Q score > 30) were obtained from the samples. Uniformity of coverage was higher than 98% in all samples (Figure 2). Reproducibility of single nucleotide variants (SNVs) and insertions/deletions (INDELs) found in the three different matrices (blood, fresh and FFPE tissues) of 7 animals was always 100%. Moreover, no statistically significant differences were observed for both total reads number and amplicon mean coverage in the three matrices (*p* > 0.05). 

**Figure 3:**
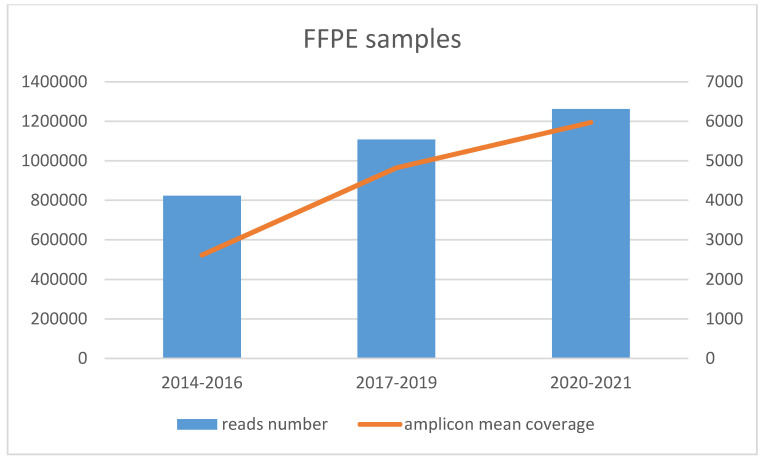
FFPE blocks grouped as old (2014–2016), medium (2017–2019), and recent (2020–2021). The output significantly improved from the ancient to the new samples, in terms of total reads number and amplicon mean coverage.

Conversely, FFPE samples stored for different numbers of years showed progressively better results from the oldest to the recent ones in terms of total reads number and amplicon mean coverage (Figure 3). These results are statistically significant with *p*-values 0.00819 and 0.00174 for total reads and coverage, respectively. 

A total of 42 different genetic variants were identified in 22 dogs. In particular, 12 exonic and 4 intronic variants were found in *BRCA1*, while 23 exonic and 3 intronic variants were identified in *BRCA2* (Table 3). According to human classification, variations in *BRCA1* were catalogued as likely benign (n. 2), and not referenced (n. 14), while mutations in *BRCA2* were classified as likely benign (n. 3), variants of unknown significance (n. 5), not referenced (n. 15), and not found in humans (n. 3).

Regarding the exonic mutations, we observed 10 coding single nucleotide polymorphisms (SNPs) and 2 synonymous SNPs in *BRCA1*, whereas in *BRCA2,* 17 variants were coding SNPs, 4 synonymous SNPs, 1 deletion and 1 insertion both of 3 bp (Table 4). 

## 4. Discussion

Amplicon sequencing (AmpliSeq) is a targeted approach which enables researchers to analyze genetic variation in specific genomic regions. The ultra-deep sequencing of PCR products allows efficient variant identification and characterization in a wide range of fields [17,18,19]. AmpliSeq is particularly useful for the discovery of rare somatic mutations in complex samples such as tumors mixed with germline DNA. Moreover, the information generated by targeted gene panels can be helpful for diagnostic classification, therapeutic decisions, and/or prognostic insights [20]. In this study, we described the development and validation of an AmpliSeq approach for the identification *BRCA1* and *BRCA2* variants in dogs (*Canis familiaris*). The Amplicon panel has been designed to cover the whole *BRCA1/BRCA2* exome and the proximal intronic regions (~30–50 bp), in both directions. The short-length PCR panel has been properly designed for fragmented DNA (e.g., FFPE samples). Indeed, despite the high degradation of DNA observed in FFPE samples, particularly true for DNA obtained from older FFPE blocks (>5 years), the assay was able to produce good quality results for all the samples. In the first sequencing attempt, we pooled together all the samples without previous individual quantification and normalization. The pool was then quantified and diluted as recommended but the resulting coverage was unbalanced between samples and sometimes unsuitable for downstream analysis. The poorer the DNA quality the lower the total reads number and amplicon mean coverage occurred. The same libraries were thus quantified and normalized at the equal molar concentration before pooling and sequencing. Following this procedure, we obtained more standardized reads number and amplicon mean coverage among the samples. Despite the variation observed in FFPE samples, depending on the time of storage, all the samples were successfully analyzed. Individual normalization is thus a critical point to obtain reliable quality results for strong fragmented DNA samples. 

Variant analysis of the seven dogs for which all three matrices were available in this study, confirmed the high reproducibility of the whole workflow, from DNA extraction to variant calling. In such samples, we could establish with certainty the germinal nature of the variants found, although this was not among our goals.

In addition to the method validation, this study also attempted the interpretation of the variants found in dogs. In humans, family information is generally integrated to evaluate the probability that sequence variants are associated with genetic predisposition [21]. In contrast, much of this information is not available for dogs. The results of dog samples were thus interpreted on the basis of software and classifications developed for humans.

In this study, the dog versus human BRCA1/BRCA2 comparison was established by using Alamut Visual Plus, while the interpretation of the dog variants found in both humans and dogs were carried out using ClinVar, a software that archives and aggregates information about relationships among variation and health in humans. Only two BRCA1 variants were referenced in humans and classified as likely benign, while eight variants of BRCA2 were also reported in humans; five were classified as of uncertain significance (UV) and three as likely benign. Among UVs, the human BRCA2 variants p.(Lys1440Arg), p.(Arg1997Lys) and p.(Trp395Cys), showed the same amino acid substitution described here in dogs, while at the position of human BRCA2 p.(His150Arg), we found p.(His143Pro). 

Four out of 22 different dogs (ID: 2, 4, 14, 15) showed c.4314A>G—p.(Lys1435Arg) together with c.4283A>C—p.(Thr1425Pro), both in exon 11 of *BRCA2* and previously described in the literature as implicated in carcinogenesis of dogs [13,14,22]. Both mutations were also confirmed by Sanger sequencing (data not shown). Of them, two showed an intraductal papillary carcinoma, one a solid carcinoma and one a complex carcinoma in a broad range of grade of malignancy (I–III); correlation to a specific histotype is thus excluded.

In healthy dogs (n = 2), we did not find c.4314A>G nor c.4283A>C in FFPE samples, nor in fresh tissues. These variations are located in the BRC3 repeat domain, which plays a fundamental role in the interaction with the protein RAD51, and affect well conserved positions [23,24] in available vertebrate species. Only one of these two dog BRCA2 variants, c.4314A>G—p.(Lys1435Arg), was also reported by ClinVar and classified in humans as of uncertain significance. We found this mutation alone in 18 out of 22 dogs (81%). Another dog BRCA2 variant, found in exon 11, c.4175G>A—p.(Arg2022Lys), was reported in humans and classified by ClinVar as Uncertain Significance. This variant was found on the mammary gland of a male dog, described as intraductal carcinoma, located in one of the BRCA repeats critical for binding to RAD51 [25,26]. Further analyses are needed to evaluate possible correlations.

Finally, it is worth considering the c.10204_10206insAAA variation that is positioned in the nuclear localization signal 2 (NLS2), which plays a key role in the mislocation of BRCA2 in humans [27]. Recent studies suggest that c.10204_10206insAAA may be associated with significantly higher mammary tumor morbidity rates in dogs [13,14]. We found this insertion in 18 out of 22 dogs (81%) in exon 27 of *BRCA2*. 

## 5. Conclusions

Canine and human tumors share numerous traits: genetic and histopathological characteristics, biological behavior, molecular targets and response to standard antineoplastic treatments. For this reason, dogs offer an extraordinary opportunity to improve our knowledge of tumor biology in both species. The AmpliSeq NGS assay developed in this study for dog *BRCA1* and *BRCA2* will help to generate reproducible and robust results. The data obtained here are part of a database of variants that will be further increased by new findings from pathologic and healthy tissues of both historical and new samples. All these data will certainly lead to a better understanding of the disease and a more careful management of health care in both humans and dogs.

## Figures and Tables

**Table 1:** Anamnestic and histological data of the dogs involved in the study.

Dog ID	Year of Collection	Sex and Hormonal Status	Breed	Age (Years)	Tissue	Histologic Type ^†^	Grade of Malignancy ^‡^
1 ^§^	2019	Fi	Yorkshire terrier	8	Mammary	Tubular carcinoma	I
2 ^§^	2019	Fi	Maremma sheepdog	11	Mammary	Intraductal papillary carcinoma (cystic type)	I
3 ^§^	2020	Fi	Mixed breed	10	Mammary	Tubular carcinoma	III
4 ^§^	2020	Fi	Mixed breed	10	Mammary	Solid carcinoma	I
5	2020	Fi	Golden retriever	9	Mammary	Intraductal papillary carcinoma (cystic type)	I
6	2021	Fi	Pinscher	15	Mammary	Tubulopapillary carcinoma	I
7 ^§^	2021	Fi	Mixed breed	12	Mammary	Anaplastic carcinoma	II
8 ^§^	2021	F-	Pinscher	17	Mammary	Tubular carcinoma, with foci of necrosis	II
9 ^§^	2021	Fi	German shepherd	12	Mammary	Ductal carcinoma	I
10	2016	Mi	Mixed breed	13	Mammary	Intraductal carcinoma	I
11	2020	Mi	Mixed breed	13	Mammary	Tubulopapillary carcinoma, with foci of necrosis	II
12	2018	Fi	German shepherd	6	Mammary	Anaplastic carcinoma with multifocal aspects of intraductal papillary carcinoma and foci of necrosis	III
13	2016	Fn	German shepherd	5	Mammary	Tubular carcinoma	I
14	2016	Fn	Mixed breed	4	Mammary	Complex carcinoma	I
15	2016	Fn	Mixed breed	5	Mammary	Intraductal papillary carcinoma (cystic type), with focal areas of squamous metaplasia	III
16	2015	Fn	American pit bull terrier	11	Mammary	Solid carcinoma with foci of necrosis	II
17	2015	Fn	Poodle	4	Mammary	Tubular carcinoma	II
18	2015	Fn	Siberian husky	-	Mammary	Solid carcinoma	II
19	2015	Fn	Golden retriever	3	Mammary	Tubulopapillary carcinoma	I
20	2014	Fi	German shepherd	9	Mammary	Intraductal papillary carcinoma	II
21	2021	Fi	Italian Cane Corso	4	Mammary	Normal tissue	n/a
22	2021	F-	German shepherd	4	Mammary	Normal tissue	n/a

Fi = Intact female; Fn = Neutered female; F- = no information regarding the hormonal status; Mi = Intact male. n/a = not applicable. ^†^ Histological classification for mammary [15]. ^‡^ Grading System for tumor mammary tissue [16] ^§^ Dogs for which all three matrices blood, FFPE blocks and fresh tissues, were used in this study.

**Table 2:** DNA quality control.

Parameters	FFPE			Blood	Fresh Tissue
	Old	Medium	Recent		
DIN	1.5–2.2	2.3–4.2	3.4–6.2	8.3–9.1	7.7–8.2
Concentration (ng/µL)	8.2–19.6	26.2–34.5	12.8–24.8	12.0–28.6	16.3–31.0

FFPE old samples: 2014–2016, FFPE medium samples: 2017–2019, FFPE recent samples: 2020–2021.

**Table 3:** Variants found in BRCA genes.

	N° Tot	N° Exonic Variants	N° Intronic Variants
*BRCA1* variants	16	12	4
*BRCA2* variants	26	23	3

**Table 4:** Details on germline and somatic variants found in the dogs enrolled in the study.

Gene	Chromosome (Dog)	Exon/Intron	Nucleotide Change ^§^	Protein Change	Human Variant at Corresponding Position ^§^	Human Clinical Classification
*BRCA1*	chr 9	INT 2	c.81-26delC	-	c.81-26delC	Not referenced
*BRCA1*	chr 9	INT 2	c.81-18delC	-	c.81-12delC	Benign/Likely benign
*BRCA1*	chr 9	INT 3	c.135-43delA	-	c.135-43delA	Not referenced
*BRCA1*	chr 9	EX 5	c.169G>A	p.(Gly57Arg)	c.169G>A; p.(Gly57Arg)	Not referenced
*BRCA1*	chr 9	EX 10	c.606A>G	p.(Asp200Gly)	c.602A>G; p.(Asp201Gly)	Not referenced
*BRCA1*	chr 9	EX 10	c.611A>G	p.(Lys202Glu)	c.607G>A; p.(Glu203Lys)	Not referenced
*BRCA1*	chr 9	EX 11	c.723G>A	p.(Gly239Ser)	c.720A>G; p.(Gln240=)	Not referenced
*BRCA1*	chr 9	EX 11	c.1315C>T	p.(Ala436Val)	c.1316C>T; p.(Ala439Val)	Not referenced
*BRCA1*	chr 9	EX 11	c.1337A>C	p.(Arg443Ser)	c.1338A>C; p.(Arg446Ser)	Not referenced
*BRCA1*	chr 9	EX 11	c.3628A>G	p.(Thr1207Ala)	c.3616G>A; p.(Ala1206Thr)	Not referenced
*BRCA1*	chr 9	EX 11	c.3963G>A	p.(Ser1318=)	c.3951G>A; p.(Leu1317=)	Likely benign
*BRCA1*	chr 9	EX 13	c.4219C>T	p.(Thr1403=)	c.4197C>T; p.(Thr1399=)	Not referenced
*BRCA1*	chr 9	INT 13	c.4381-19C>T	-	c.4358-16C>T	Not referenced
*BRCA1*	chr 9	EX 16	c.4777C>T	p.(Ser1588Pro)	c.4754C>T; p.(Pro1585Leu)	Not referenced
*BRCA1*	chr 9	EX 19	c.5203G>A	p.(Arg1729Lys)	c.5177G>A; p.(Arg1726Lys)	Not referenced
*BRCA1*	chr 9	EX 24	c.5525 G>A	p.(Ala1869Thr)	c.5581A>G; p.(Ser1861Gly)	Not referenced
*BRCA2*	chr 25	EX 4	c.308T>C	p.(Ile103Thr)	c.326T>C; p.(Val109Ala)	Not referenced
*BRCA2*	chr 25	INT 4	c.408-9_408-8del	-	-	Not found
*BRCA2*	chr 25	EX 5	c.428A>G	p.(His143Pro)	c.449A>G; p.(His150Arg)	Uncertain significance
*BRCA2*	chr 25	INT 5	c.455-40del	-	-	Not found
*BRCA2*	chr 25	EX 10	c.1122C>T	p.(Thr371Ile)	c.1139G>T; p.(Ser380Ile)	Not referenced
*BRCA2*	chr 25	EX 10	c.1168T>G	p.(Cys386Trp)	c.1185G>T; p.(Trp395Cys)	Uncertain significance
*BRCA2*	chr 25	EX 11	c.1937G>A	p.(Glu643Lys)	c.1957G>A; p.(Glu653Lys)	Not referenced
*BRCA2*	chr 25	EX 11	c.2131T>C	p.(His707=)	c.2148G>C; p.(Gln716His)	Uncertain significance
*BRCA2*	chr 25	EX 11	c.2154A>C	p.( Pro715 Gln)	c.2170A>C; p.(Lys724Gln)	Not referenced
*BRCA2*	chr 25	EX 11	c.2164C>A	p.(Ser718=)	c.2181A>C; p.(Ser727=)	Likely benign
*BRCA2*	chr 25	EX 11	c.2193C>T	p.(Ala728Val)	c.2210C>T; p.(Ala737Val)	Not referenced
*BRCA2*	chr 25	EX 11	c.2232A>G	p.(Asp741Ser)	c.2246G>A; p.(Ser749Asn)	Not referenced
*BRCA2*	chr 25	EX 11	c.2269A>C	p.(Lys801Gln)	c.2431A>C; p.(Lys811Gln)	Not referenced
*BRCA2*	chr 25	EX 11	c.4283A>C	p.(Thr1425Pro)	c.4288A>C; p.(Thr1430Pro)	Not referenced
*BRCA2*	chr 25	EX 11	c.4314A>G	p.(Lys1435Arg)	c.4319A>G; p.(Lys1440Arg)	Uncertain significance
*BRCA2*	chr 25	EX 11	c.4147G>A	p.(Val2013Ile)	c.5962G>A; p.(Val1988Ile)	Likely benign
*BRCA2*	chr 25	EX 11	c.4175G>A	p.(Arg2022Lys)	c.5990G>A; p.(Arg1997Lys)	Uncertain significance
*BRCA2*	chr 25	EX 11	c.4198G>A	p.(Asp2030Asn)	c.6013G>A; p.(Asp2005Asn)	Not referenced
*BRCA2*	chr 25	EX 11	c.4204G>A	p.(Ala2032Thr)	c.6019A>G; p.(Thr2007Ala)	Not referenced
*BRCA2*	chr 25	EX 12	c.6952_6954del	p.(Leu2306 o 2307del)	c.6868_6870del; p.(Leu2290del)	Not referenced
*BRCA2*	chr 25	EX 12	c.6966C>T	p.(Phe2310=)	c.6879T>C; p.(Phe2293=)	Not referenced
*BRCA2*	chr 25	EX 16	G>A	p.(Asp2611Asn)	c.7774G>A; p.(Asp2592Asn)	Not referenced
*BRCA2*	chr 25	EX 18	T>C	p.(Ile2683=)	c.7992T>C; p.(Ile2664=)	Likely benign
*BRCA2*	chr 25	INT 25	c.9692+57A>T	-	-	Not found
*BRCA2*	chr 25	EX 27	c.10204_1026insAAA	p.(Met3332_Ile3333insLys)	c.9936_9938dup; p.(Lys3315dup)	Not referenced
*BRCA2*	chr 25	EX 27	c.10450A>C	p.(Thr3405Pro)	c.101111A>C; p.(Thr3371Pro)	Not referenced

^§^ prefix “c.” for cDNA sequence; the nucleotide number; a wild-type nucleotide; the symbol “>” (indicating a change), the prefix “del.” (indicating deletion) or “ins.” (indicating insertion); and the mutant nucleotide http://www.hgvs.org/mutnomen/recs-DNA.html (accessed on 27 May 2022). The DNA sequence numbering is based on the cDNA sequence of *BRCA1/2* (NCBI RefSeq NM_001013416.1 and NM_001006653.4, respectively) following the recommendations of the Human Genome Variation Society (first position of the translation initiation codon ATG c.1). prefix “p.” for protein sequence; reference amino acid (three-letter code); position; amino acid change (three-letter code) or “fs” prefix (indicating frameshift) http://www.hgvs.org/mutnomen/recs-prot.html (accessed on 27 May 2022).

## Data Availability

Data are available upon request to the corresponding author.

## Supplementary Materials

The following supporting information can be downloaded at: https://www.mdpi.com/article/10.3390/life12060851/s1. Custom amplicons and manifest file.
